# Oral Health Knowledge and Practices of Secondary School Students, Tanga, Tanzania

**DOI:** 10.1155/2011/806258

**Published:** 2011-11-17

**Authors:** Lorna Carneiro, Msafiri Kabulwa, Mathias Makyao, Goodluck Mrosso, Ramadhani Choum

**Affiliations:** ^1^Department of Restorative Dentistry, Muhimbili University of Health and Allied Sciences, P.O. Box 65451, Dar es Salaam, Tanzania; ^2^Department of Preventive and Community Dentistry, Muhimbili University of Health and Allied Sciences, P.O. Box 65014, Dar es Salaam, Tanzania; ^3^School of Dentistry, Muhimbili University of Health and Allied Sciences, P.O. Box 65014, Dar es Salaam, Tanzania

## Abstract

A good quality of life is possible if students maintain their oral health and become free of oral disease. A structured questionnaire assessed 785 students' level of oral health knowledge and practices. About 694 (88.4%) students had adequate level of knowledge on causes, prevention, and signs of dental caries, 760 (96.8%) on causes and prevention of periodontal diseases, 695 (88.5%) on cigarette smoking as cause of oral cancer, and 770 (98.1%) students on importance of dental checkups. Majority 717 (91.3%) had adequate practice of sugary food consumption; while 568 (72.4%) had acceptable frequency of tooth brushing, 19 (2.4%) brushed at an interval of twelve hours, and 313 (39.9%) visited for checkup. Majority of students had an adequate level of knowledge on oral health but low level of oral health practices. Both genders had similar level of knowledge with male predominance in oral health practices. Age had no influence on the level of oral health knowledge and practices of students.

## 1. Introduction

Oral health as an essential aspect of general health can be defined as “a standard of health of the oral and related tissues which enables an individual to eat, speak, and socialize without active disease, discomfort or embarrassment and which contributes to general well-being” [1]. Oral health knowledge is considered to be an essential prerequisite for health-related practices [2], and studies have shown that there is an association between increased knowledge and better oral health [3, 4]. Those who have assimilated the knowledge and feel a sense of personal control over their oral health are more likely to adopt self-care practices [5]. 

The Policy guidelines for Oral Health Care in Tanzania (2002) aim at improving the oral health of Tanzanians with focus on those most at risk by sensitizing communities on preventable oral health problems [6]. Part of the Essential Package of Oral Health Care in Tanzania includes prevention of oral diseases through provision of oral health education in primary schools, at the Reproductive and Child Health Clinics (RCH), and the general public [7].

Oral health education has been part of the primary school curriculum in Tanzania since 1982 and implemented by teachers at primary schools [6]; however, the oral health education sessions addressed oral hygiene by lectures, and it was observed to be deficient in content and in methods [8]. To address the noted deficiency, a simple oral health education manual was designed to answer the educational needs of the pupils, and using it as a framework, sessions taught both the concepts and the skills of oral health care in a manner that actively involved the pupils in the learning process [9], and it was shown that pupils actively studied the concepts and practical skills for dietary choices and tooth brushing [10]. The district local governments have a basket fund that can be used to promote oral health education programs among the priority groups like RCH clinics and the community as a whole including secondary school students. It is the responsibility of the dental personnel at regional, district, and health centres to ensure adequate deliverance of oral health education [9].

The level of oral health knowledge and practices of secondary school students is unknown and worthy of investigation, and this study aimed at assessing the level of oral health knowledge and practices of secondary school students in Tanga, Tanzania. 

## 2. Study Population and Methods

This cross-sectional study conducted between September and November, 2010, assessed the level of oral health knowledge and practices of 785 secondary school students in Tanga Region, Tanzania. Tanga Region has eight districts, namely, Tanga, Lushoto, Korogwe, Muheza, Mkinga, Pangani, Handeni, and Kilindi, from which two districts, namely Tanga and Lushoto, were conveniently chosen for this study.

Records show that the total student population of secondary schools in Tanga district is 16,993 of which males are 9,573 and females are 7,420, while that of Lushoto district is 26,573 of which males are 12,983 and females are 13,590.

A total of eight public secondary schools, four from Lushoto district and four from Tanga district, were conveniently chosen for the study. From each school, a sample size of hundred students, fifty students from form II (25 boys and 25 girls) and fifty students from form III (25 boys and 25 girls) were randomly selected by the teachers on duty. Estimated sample size was 800 students with a response rate of about 98%, as questionnaires of 15 students incorrectly filled were omitted from analysis. Form I students were excluded intentionally as knowledge from primary schools may influence obtained results and form IV because of their involvement in the end of school examinations.

Following written consent, a self-administered questionnaire that was translated from English into Kiswahili (local language) assessed level of oral health knowledge and practices of students. Oral health knowledge was assessed using 9 questions; 4 questions assessed knowledge on dental caries (frequent consumption of sugary foods can cause tooth decay; tooth decay can be prevented by using fluoridated toothpaste twice a day; tooth decay can be prevented by controlling frequent consumption of sugary foods; presence of a cavity in a tooth indicates tooth decay); 3 questions assessed knowledge on periodontal disease (ineffective tooth brushing can cause gum disease, effective tooth brushing can prevent gum disease; bleeding during brushing may indicate presence of gum disease); one question assessed knowledge on relationship between smoking and oral cancer (smoking cigarettes and chewing tobacco for a long time may result in oral cancer), and the last question assessed knowledge on the importance of dental checkup (early occurrence of oral diseases can be diagnosed by seeking dental services regularly). Responses to the above questions were either “yes,” “no,” or “I don't know.” For analysis, the “no” and “I don't know” responses were coded as “no = 0” and the “yes = 1.” A student was regarded as knowledgeable if more than half the number of questions in that category was correctly answered and was regarded not knowledgeable if less than half of the questions were correctly answered. 

Using continuous response scales oral health practices were assessed using 7 questions; four questions assessed number of times a day oral hygiene is practiced (once a day, twice, or more times a day) and time of practicing oral hygiene (before or after breakfast, after lunch, or before going to bed at night), frequency of consumption of sugary foods or drinks (1-2 times per day, 3-4 times per day, and 5 or more times per day), and practice of dental visits for checkup was assessed using a dichotomous scale (yes or no). Using a categorical scale, the remaining 3 questions assessed items used to practice oral hygiene (plastic tooth brush or wooden tooth brush (mswaki), additional devices used to clean teeth (dental floss, toothpicks, and others) and additives used to clean teeth (toothpaste, charcoal, salt, or sand). 

Responses to the number of times a day oral hygiene was practiced were coded as adequate practice = 0 (if practice was twice, thrice, or more than three times a day) and inadequate practice = 1 (if practice was once a day). Response to the time of practicing oral hygiene was coded as adequate practice = 0 (if brushing was before or after breakfast and before going to bed at night) and inadequate practice = 1 (if brushing was done only before breakfast, only after breakfast, only after lunch, or only before going to bed at night). Frequency of consumption of sugary foods or drinks was coded as adequate practice = 0 (if less than five times a day) and inadequate practice = 1 (if five or more times a day). Responses to the practice of having ever visited a dentist for checkup was coded as adequate practice = 0 (if a student responded “yes”) and inadequate practice = 1 (if a student responded “no”). 

Data collected was coded, and frequency distributions and Chi-square tests were used to determine the level of statistical significant difference which was set at *P* < 0.05. Ethical clearance was obtained from the Director of Research and Publications, Muhimbili University of Health and Allied Sciences, P.O. Box 65001, Dar es salaam, Tanzania.

## 3. Results

A total of 785 secondary school students with an age range of 14 to 22 years and mean age of 16.9 years participated in the study. As shown in Table 1, there were slightly more students in Tanga district (*n* = 394; 50.2%)  compared to Lushoto district (*n* = 391; 49.8%), and females (*n* = 395; 50.3%) were more than males. Majority (*n* = 548; 69.8%) of the students belonged to the 14–17 years age group.

More than three quarters (*n* = 694; 88.4%) of the study population had adequate knowledge on causes, prevention and signs of dental caries. Students in Tanga district showed no statistical significant difference between age or sex, while in Lushoto district, there was a statistical significant difference between sex (*P* < 0.05) but no statistical significant difference between the age groups. Nearly all (*n* = 760; 96.8%) of the students had adequate knowledge on causes and prevention of periodontal diseases, about ninety percent (*n* = 695; 88.5%) of participants had adequate knowledge on cigarette smoking as cause of oral cancer, and nearly all (*n* = 770; 98.1%) participants had adequate knowledge on importance of dental checkups; however, there was no statistical significant difference observed between districts, age or sex (Table 2). 

As shown in Table 3, majority (*n* = 717; 91.3%) of the study population in both districts reported to have an acceptable level of practice of frequency of sugary food consumption. In Tanga district, males with acceptable practice were significantly more than females (*P* < 0.05); however, no statistical significant difference was observed between the age groups, while in Lushoto district, no statistical significant difference was observed between age and sex. The number of students who had an acceptable level of practice of frequency of brushing teeth (twice or more times a day) were 568 (72.4%). There was no statistical significant difference between age and sex in Tanga district, but there were statistically significantly more females than males in Lushoto who brushed twice or more times a day (*P* < 0.01). Only 19 (2.4%) of subjects had an acceptable practice of brushing teeth at an interval of twelve hours, and no statistical significant difference was observed either in Tanga or Lushoto districts between age and sex. About 313 (39.9%) students reported acceptable level of practice of dental visits for checkup. There was no significant statistical difference between age and sex in Tanga district, while there were more males than females in Lushoto district who visited a dental clinic (*P* < 0.01). 

The practice of maintaining oral hygiene was reported by 784 students, and majority used the plastic tooth brush (*n* = 754; 96%) compared to the wooden tooth brush (mswaki). The 30 (4%) students using the wooden tooth brush (mswaki) were mainly from Lushoto district. Of the 785 who reported the practice of using additional items to aid cleaning, majority (*n* = 664; 84.6%) of the students used tooth paste; however, some used charcoal (*n* = 86; 11.0%) and salt (*n* = 34; 4.3%). Majority of the students (*n* = 770; 98.1%) reported the use of other items for cleaning teeth like tooth picks (*n* = 636; 81%), match sticks (*n* = 22; 2.8%), finger nails (*n* = 14; 1.8%), dental floss (*n* = 89; 11.3), leaves (*n* = 7; 0.9%), and pins (*n* = 2; 0.3%).

## 4. Discussion

Oral health education is part of the curriculum of primary schools and its delivery in secondary schools is supposed to be done by dental personnel at regional, district, and health centres. This study assessed the level of oral health knowledge and practices of secondary school students in Tanga Region, Tanzania. The cross-sectional study design took into consideration accessibility to the target group, time factor, merger source of manpower, and funds. The limitation of using a convenient sampling method used in this study is acknowledged, and although regarded to be nonrepresentative of the total population, it gives a reflection to the real picture of the general population, and we have no reason to believe that the sample taken was in any way very different from the rest of the population. Bias may have been introduced from the false-positive responses of participants to the self-administered questionnaire, as some students may have copied responses or had prior exposure to oral health knowledge. 

Results from this study revealed a high proportion of students with adequate level of knowledge on oral health, and these findings are similar to those reported in other studies done in Tanzania, [11, 12] and Kuwait [13]. This could be a result of either oral health knowledge that they might have learnt while at primary school or acquired through the media or an outcome of wanting to please the researchers. Most students' level of knowledge on causes, prevention, and signs of dental caries and of periodontal disease was adequate and in accordance to findings that have been reported in Tanzania [11] and Jordan [14]; however they are different from those reported in Sweden [15] and Kuwait [16]. The reportedly higher proportion of students with adequate knowledge on causes, prevention, and signs of dental caries and of periodontal disease depicts that students can retain and recall the acquired knowledge as they grow. 

Similar to findings from this study, a high proportion of students with adequate level of knowledge on cigarette smoking as a cause of oral cancer were also reported in Kenya [17] and in UK [18]. It is possible that this knowledge was obtained from the media rather than from oral health education sessions taught in schools, as the oral health curriculum in primary schools do not include knowledge on cigarette smoking as a cause of oral cancer. Most of the students had adequate knowledge on importance of regular dental visits, and although this may be the truth, Kikwilu et al. [19] reported that only a quarter of those who experienced oral pain or discomfort sought emergency oral care from oral health care facilities. Gómez et al. [20] in their report highlight on the importance of early detection as a cornerstone to improve survival. 

With the exception of females from Lushoto being more knowledgeable than males on knowledge on causes, prevention, and signs of dental caries, there was gender equality in oral health knowledge among secondary school students similar to what was observed in a study conducted in Khartoum Province, Sudan [21].

Nearly all of the students had an acceptable level of practice on frequency of sugary food consumption as recommended by the recent systematic analysis that free (added) sugar should remain below 10% of the energy intake and the consumption of food/drinks containing free sugars should be limited to a maximum of four times per day [22]. Contrary to the reported findings, Masalu et al. [23] found that female students were more likely to believe in the importance of limited sugar consumption than their male counterparts. The observed low consumption of sugary foods and drinks by students could be related to the low socioeconomic status of parents which may render the consumption of sugary foods or drinks a luxury. Certain oral diseases, such as chronic periodontitis and caries that are considered public health problems may be alleviated by effective and regular self tooth brushing [24]. Twice-a-day tooth brushing seems to be an established practice in several industrialized countries [25–28], and majority of the research participants practiced twice-a-day tooth brushing. In Tanzania, other studies have reported once-a-day tooth brushing by their participants [23, 29], and similar findings were also reported in Iran [30] and Thailand [31]. Although twice-a-day tooth brushing has been reported, only 2.4% of students had an acceptable practice of brushing teeth at an interval of twelve hours as recommended showing that students are not informed on the importance of brushing twice a day at an interval of twelve hours. Only a few of the students had acceptable level of practice of attending dental clinics for checkup, and a similar low attendance was reported by other researchers in Tanzania [19, 32] and Nigeria [33]. In most instances, practices performed by parents are handed over to children, and in Tanzania, dental visits for checkup has not been a practice, and most people only attend dental clinic when they experience pain [19].

In accordance with findings from another study done in Tanzania [34] and Nigeria [33], the plastic toothbrushes were commonly used and preferred to the wooden tooth brush (mswaki). However, in Zimbabwe [35] and Kenya [36], the mswaki was the most commonly used cleaning device in comparison to the plastic toothbrush. The use of the plastic toothbrush by students in this study may be related to previous oral health education sessions, whereby the plastic toothbrush was used for demonstration or merely because of one wanting to be modern. The use of mswaki as depicted by few could also be related to parents' socioeconomic status or religious influence. The efficacy of the mswaki in maintaining oral hygiene in regard to the plastic toothbrush still needs to be investigated [37]. Many participants used tooth paste to aid oral hygiene, and this finding was similar to that reported by Mwakatobe and Mumghamba [38] and Nyandindi et al. [34]; however, the reported use of charcoal and salt by few participants could be related to socioeconomic factors of parents or traditional beliefs that they assist in improving ones oral health status. 

## 5. Conclusion

Majority of students had an adequate level of knowledge on oral health but low level of oral health practices. Both genders had similar level of knowledge with male predominance in oral health practices. Age had no influence on the level of oral health knowledge and practices of students.

## 6. Recommendations

- The oral health teaching manual used should be revised to include newer concepts of oral health care like twice a day tooth brushing at an interval of twelve hours using fluoride toothpaste, cigarette smoking as a cause of oral cancer and importance of regular dental visits for checkup.
- Oral health knowledge and practices should be taught to secondary school students.
- More studies should be conducted in other regions for comparison.

## Figures and Tables

**Table 1:** Distribution of study participants by district, age, and sex (*N* = 785) (percentages shown in parenthesis).

District	Age group (years)	Sex					
		Male		Female		Total	
		*n*	%	*n*	%	*n*	%
Tanga	14–17	120	(62.2)	163	(81.1)	283	(71.8)
	18+	73	(37.8)	38	(18.9)	111	(28.2)

Lushoto	14–17	121	(45.7)	144	(54.3)	265	(67.8)
	18+	76	(60.3)	50	(39.7)	126	(32.2)

Total		390	(49.7)	395	(50.3)	785	(100.0)

**Table 2:** Distribution of student's with adequate level of oral health knowledge by district, age, and sex (percentages shown in parenthesis).

Question	Tanga district								Lushoto district							
	Sex				Age (years)				Sex				Age (years)			
	Male		Female		14–17		18+		Male		Female		14–17		18+	
	*n*	%	*n*	%	*n*	%	*n*	%	*n*	%	*n*	%	*n*	%	*n*	%

Knowledge on causes, prevention and signs of dental caries	176	(91.2)	173	(86.1)	250	(88.3)	99	(89.2)	166	(84.3)	179	(92.3)*	237	(89.4)	108	(85.76)

Knowledge on causes and prevention of periodontal diseases	188	(97.4)	194	(96.5)	272	(96.1)	110	(99.1)	189	(95.9)	189	(95.9)	258	(97.4)	120	(95.2)

Knowledge on cigarette smoking as cause of oral cancer	177	(91.7)	180	(90.0)	253	(89.7)	104	(93.7)	172	(88.2)	166	(86.0)	227	(86.6)	111	(88.1)

Knowledge on importance of dental checkups	191	(99.0)	198	(99.0)	278	(98.6)	111	(100)	193	(98.0)	188	(97.4)	259	(97.7)	122	(97.6)

Chi-square test: *P < 0.05.

**Table 3:** Distribution of students with acceptable oral health practices by district, age, and sex (percentages shown in parenthesis).

Question	Tanga district								Lushoto district							
	Sex				Age (years)				Sex				Age (years)			
	Male		Female		14–17		18+		Male		Female		14–17		18+	
	*n*	%	*n*	%	*n*	%	*n*	%	*n*	%	*n*	%	*n*	%	*n*	%

Consuming sugary foods less than five times a day	155	(89.6)*	152	(81.3)	215	(83.3)	92	(90.2)	161	(92.0)	164	(90.1)	220	(90.5)	105	(92.1)

Brushing teeth two or more times a day	130	(68.1)	152	(76.0)	197	(69.9)	85	(78.0)	130	(66.3)	156	(80.4)**	194	(73.5)	92	(73.0)

Brushing teeth at an interval of 12 hours	2	(1.0)	9	(4.5)	9	(3.2)	2	(1.8)	3	(1.5)	5	(2.6)	5	(1.9)	3	(2.4)

Ever visited a dental clinic	81	(42.0)	96	(47.8)	121	(42.8)	56	(50.5)	82	(41.6)**	54	(27.8)	92	(34.7)	44	(34.9)

Chi-square test: *P < 0.05; **P < 0.01.
